# Early changes of the kinetics of monocyte trem-1 reflect final outcome in human sepsis

**DOI:** 10.1186/s12865-014-0063-y

**Published:** 2014-12-23

**Authors:** Androniki Marioli, Marina Koupetori, Maria Raftogiannis, Maria Patrani, Nikolaos Antonakos, Maria Pavlaki, Georgios Adamis, Georgia Dougekou, Georgia Damoraki, Iraklis Tsangaris

**Affiliations:** 2nd Department of Internal Medicine, Sismanogleion General Hospital, 15126 Athens, Greece; 1st Department of Internal Medicine, Thriasio General Hospital, 19600 Elefsis, Greece; 4th Department of Internal Medicine, Medical School, University of Athens, 12462 Athens, Greece; Intensive Care Unit, Korgialeneion-Benakeion Hospital, 11526 Athens, Greece; Department of Internal Medicine, Argos General Hospital, 21200 Argos, Greece; 1st Department of Internal Medicine, G.Gennimatas General Hospital, 11527 Athens, Greece; 2nd Critical Care Department, “Attikon” University Hospital, 1 Rimini Str., Athens, 12462 Greece

**Keywords:** Sepsis, Outcome, TREM-1, sTREM-1, Monocytes

## Abstract

**Background:**

TREM-1 (triggering receptor expressed on myeloid cells), a receptor expressed on neutrophils and monocytes, is upregulated in sepsis and seems to tune the inflammatory response. We explored the expression of TREM-1 at the gene level and on cell membranes of monocytes and association with clinical outcome.

**Methods:**

Peripheral venous blood was sampled from 75 septic patients (39 patients with sepsis, 25 with severe sepsis and 11 with septic shock) on sepsis days 1, 3 and 7. TREM-1 on monocytes was measured by flow cytometry; gene expression of *TREM-1* in circulating mononuclear cells was assessed by real-time PCR. sTREM-1 was measured in serum by an enzyme immunoassay.

**Results:**

Although surface TREM-1, sTREM-1 and *TREM-1* gene expression did not differ between sepsis, severe sepsis and septic shock on day 1, survivors had greater expression of surface TREM-1 on days 3 and 7 compared to non-survivors. sTREM-1 on non-survivors decreased on day 3 compared to baseline. Patients with increase of monocyte gene expression of *TREM-1* from day 1 to day 3 had prolonged survival compared to patients with decrease of gene expression of *TREM-1* from day 1 to day 3 (p: 0.031).

**Conclusions:**

Early decrease of gene expression of *TREM-1* in monocytes is associated with poor outcome. A reciprocal decrease of the pro-inflammatory surface receptor TREM-1 linked with sepsis-induced immunosuppression may be part of the explanation.

**Electronic supplementary material:**

The online version of this article (doi:10.1186/s12865-014-0063-y) contains supplementary material, which is available to authorized users.

## Background

Sepsis is among the leading causes of death worldwide and a huge burden for any healthcare system. The incidence seems to be constantly increasing whereas recent reports on mortality vary considerably [1]. Epidemiological data coming from Australia and New Zealand show a stable decrease of mortality by severe sepsis from 35.0% in 2000 to 18.4% in 2012 [2] whereas overall mortality from septic shock in North America from 1989 to 2008 ranged between 42.0% and 84.5% depending on the underlying infectious cause [3]. So far every therapeutic attempt to modulate the immunologic response in sepsis has been clinically unsuccessful, probably as a result of an incomplete understanding of sepsis pathophysiology [4,5].

The triggering receptor expressed on myeloid cells (TREM-1) is a pattern-recognition receptor expressed on neutrophils and monocytes [6], playing a key-role in the septic process [7]. In sepsis the expression of TREM-1 is initially up-regulated by bacterial or fungal products leading to the production of pro-inflammatory cytokines, mainly of tumor necrosis factor-alpha and of interleukin-8 [8]. The cross-talk between TREM-1 and Toll-like receptors *(*TLRs*)* seems to affect final outcome [9]. In vivo silencing of TREM-1 using siRNA duplexes resulted in increased mortality in a peritonitis mouse model, while it was protective from experimental endotoxemia [10].

The soluble counterpart of TREM-1, namely sTREM-1, is increased in septic shock and it is considered a surrogate marker than can differentiate an infectious from a non-infectious systemic inflammatory reaction particularly when this is associated with bacteremia [11]. Some publications suggest sTREM-1 as a valuable prognostic biomarker since concentrations in serum greater than 180 pg/ml are associated with unfavorable outcome [12,13].

It is widely known that the initial pro-inflammatory response of the host to a septic insult is followed by a second phase of immunosuppression characterized by re-programming of leukocytes and decrease of the expression of most pro-inflammatory genes [14]. In a recent study in a Greek population of patients with severe sepsis/shock, the gene expression *of TREM-1* in monocytes was not increased compared to healthy volunteers, while serum levels of sTREM-1 (soluble TREM-1) were increased [15]; still the time course and the clinical significance of these alterations are not clear. The objective of this study is to describe the pattern of TREM-1 expression on monocytes both on the surface and on the gene level and to evaluate its impact on the outcome of sepsis.

## Methods

### Study design

The study protocol and the informed consent form were submitted and approved by the Ethics committees of ATTIKON University Hospital and by the Ethics Committee of Sismanogleion General Hospital before start of the study. The anonymity of data was secured by a coding procedure so that each participating patient had a code consisting of a capital and a consecutive number. Written consent was provided from patients or their first-degree relatives for patients unable to consent. None of the enrolled patients participated in a former study published by the Hellenic Sepsis Study Group [16].

*Inclusion criteria*: a) age ≥ 18 years; b) sepsis due one of the following infections: community-acquired pneumonia (CAP), acute pyelonephritis, intrabdominal infection, primary bacteremia, ventilator-associated pneumonia (VAP) and health-care associated pneumonia (HCAP).

*Exclusion criteria*: a) HIV infection; b) neutropenia, defined as less than 1,000 neutrophils/mm^3^; c) organ transplantation; and d) need for blood transfusions the first 4 days of study inclusion.

Patients were classified according to the criteria of the ACCP/SCCM as uncomplicated sepsis, severe sepsis or septic shock [17].

*Acute infection of the lower respiratory tract* was defined by all of the following [18]: a) core temperature > 38°C or <36°C; b) at least two physical signs compatible with a lower respiratory tract infection like dyspnea, purulent sputum, auscultatory rales; and c) pulmonary infiltrates on chest x-ray. This was divided into CAP for patients without any recent history of hospitalization or residence to any long-term care facility the last 90 days and into HCAP for patients with history of contact with the hospital environment the last 90 days.

*Acute pyelonephritis* was defined by all of the following [19]: a) core temperature > 38°C; b) ≥ 10 white blood cells in centrifuged urine sample or ≥ 2 + in urine stick for white blood cells and nitrite; c) costovertebral angle tenderness; and d) radiological evidence consistent with the diagnosis.

*Acute intra-abdominal* infection was defined by all the following [20]: a) core temperature > 38°C or <36°C; b) abdominal tenderness; and c) radiological evidence consistent with an acute abdominal infection (abdominal x-ray, abdominal ultrasound, abdominal or computed tomography).

*Primary bacteremia* was defined by all the following [20]: a) peripheral blood culture positive for Gram-positive or Gram-negative bacteria. Coagulase-negative *Staphylococcus* spp and skin commensals were considered contaminants unless isolated at least two times or isolated from both a peripheral vein and a central catheter and they had the same antibiogram; and b) absence of any primary site of infection.

*Ventilator-associated pneumonia (VAP)* was defined for every patient under intratracheal intubation and mechanical ventilation for ≥ 48 hours by all the following [21]: a) core temperature > 38°C or < 36°C; b) purulent tracheobronchial secretions; and c) new pulmonary infiltrates on chest x-ray.

### Patients' follow up

Volumes of 14 ml of blood were sampled within twenty four hours upon enrolment of the patient in the study and then on days 3 and 7. From this amount: a) 3 ml were collected into one EDTA coated tube for flow-cytometry; b) 8 ml were collected into one heparin-coated tubes for measurement of gene expression; and c) 3 ml were collected into one pyrogen-free tubes. This last tube was centrifuged and serum was stored at −80°C. All samples were transported within one hour via a courier service to the central lab located at the 4^th^ Department of Internal Medicine, ATTIKON General Hospital. A similar amount of blood was collected from 10 healthy volunteers.

Demographic and clinical data were recorded on study enrolment. Acute Physiology and Chronic Health Evaluation (APACHE) II score was calculated at study enrolment. Appropriateness of administered antimicrobial therapy was judged based on the antibiograms of pathogens isolated from biological specimens collected on the first day of signs of sepsis. This was considered appropriate when patients were administered at least one antimicrobial active against the isolated pathogen according to the antibiogram. This was an observational study and changes of the kinetics of TREM-1 and *TREM-1* on consecutive measurements were not used to modify administered treatment. Outcome was assessed at 28 days either by the patients’ records or by telephone calls whether necessary.

### TREM and sTREM-1 measurements

#### Flow cytometry

Whole blood was stained with the monoclonal antibody anti-TREM-1 (clone 193015, R&D Systems, Minneapolis, USA) (10 μl antibody plus 50 μl WBC) at the fluorochromephycoerythrin (emission 575 nm). After incubation for 45 min at 4°C in the dark red blood cells were lysed with VersaLyse Lysing Solution (Beckaman Coulter, Immunotech, Marseille, France). The remaining white blood cells were washed after lysing with PBS (pH. 7.2) (Merck, Darmstadt, Germany). After reconstitution with 0.5 ml PBS cells were analyzed through a CYTOMICS FC-500 flow cytometer (Beckman Coulter Co, Miami, FL, USA) with gating for monocytes based on their characteristic forward and side scattering. Isotypic negative controls (IgG1) at the fluorocolour phycoerythrin were applied before the start of analysis for every patient. To verify that the gated population of monocytes had purity more than 99%, cells were stained with anti-CD3 at the fluorochrome FITC (clone UCHT1, Immunotech), anti-CD19 at the fluorochrome PE (clone J4.119, Immunochem) and anti-CD45 at the fluorochrome PC5 (clone J33, Immunotech). Results were expressed as % of gated cells and as mean fluorescence intensity (MFI). To investigate if the process of red blood cell lysis can affect results, some samples were analyzed before and after preparation.

#### Measurement of TREM-1 mRNA by real-time qPCR

Heparinized venous blood was layered over FicollHypaque (Biochrom, Berlin, Germany) and centrifuged for 20 minutes at 1400 g. Separated peripheral blood mononuclear cells (PBMCs) were washed 3 times with ice-cold phosphate buffered saline (pH: 7.2) (Biochrom) and counted in a Neubauer chamber. Their viability was more than 99% as assessed by trypan blue exclusion of dead cells. A total of 2 × 10^6^ to 5 × 10^6^ PBMCs per patient were lysed with Trizol (Invitrogen, Karlsruhe, Germany) and kept at −80°C until extraction of RNA.

RNA was extracted with chloroform (AppliChem GmbH, Darmsatdt, Germany) and gradient centrifugation for 15 minutes at 4°C and 12 000 g followed by treatment for 30 minutes at 37°C with 0.04 U/μL of DNAase (New England BioLabs, Ipswich, Massachussets). The complementary DNA (cDNA) was obtained by a reverse transcription kit according to the recommendations of the manufacturer (iScript™ cDNA Synthesis Kit, BioRad, Hercules, LA, USA) and incubated for 5 min at 25°C; 30 min at 42**°**C; and 5 min at 85**°**C. Samples treated without the addition of reverse transcriptase were used as blanks. cDNA was kept at −80°C until assayed. Expression of mRNA was assessed by the iQ™5 Cycler system (BioRad) at final volumes of 20 μl using 0.1 mg/ml of sense and antisense primers and 10 μl of FluoCycle™ II SYBR Master Mix 1X- (EuroClone S.p.A, Italy). Primers for *TREM-1* were: forward 5΄ - TGG TCT TCT CTG TCC TGT TTG −3΄ and reverse 5΄ - ACT CCC TGC CTT TTA CCT C −3΄; and for *β2-microglobulin*: forward 5′-ATG AGT ATG CCT GCC GTG TG-3′ and reverse 5′-CCA AAT GCG GCA TCT TCA AAC-3′. Conditions for PCR have already been published [11]. The number of gene copies was measured by the PFAFFL equation using gene expression coming from isolated monocytes of healthy volunteers.

#### sTREM-1 measurements

sTREM-1 was measured in serum in duplicate by an enzyme immuosorbent assay (R&D Minneapolis Mo). The lower limit of detection was 15.1 pg/ml. The co-efficient of variation of the assay was 7.65%.

#### Study endpoints

The primary study endpoint was how changes of the kinetics of TREM-1 on monocytes and of gene expression of *TREM-1* between days 1 and 3 are related with 28-day outcome. The secondary study endpoints were a) comparisons of TREM-1 on monocytes, of sTREM-1 in serum and of gene expression of *TREM-1* between the three stages of sepsis; and b) comparisons of TREM-1 on monocytes, of sTREM-1 in serum and of gene expression of *TREM-1* between survivors and non-survivors on each day of sampling.

#### Statistical analysis

Results were expressed as means ± SE. For the primary endpoint, changes of *TREM-1* gene expression between days 1 and 3 were calculated for each enrolled patient. Patients were divided into two groups of *TREM-1* gene expression: those where expression was increased from day 1 to day 3 and those where expression was decreased from day 1 to day 3. Survival of these two groups was assessed by Kaplan-Meir analysis; comparisons were done by the log-rank test. Comparison of the appropriateness of antimicrobials between these two groups was done by the Fischer’s exact test. Changes of the expression of TREM-1 on monocytes between patients with increased or decreased *TREM-1* gene expression were compared by the Mann–Whitney U test. Changes of the expression of TREM-1 on monocytes between days 1 and 3 between survivors and non-survivors were compared by the Mann–Whitney U test.

For the secondary endpoints, comparisons of *TREM-1* transcripts, of sTREM-1 and of TREM-1 on cell membranes between sepsis, severe sepsis and septic shock on day 1 were done by the Kruskal Wallis test. Comparisons between survivors and non-survivors on each day of sampling were done by the Mann- Whitney U test. Comparisons of each measured parameters on consecutive days were also done separately for survivors and for non-survivors by the Wilcoxon’s test. Any value of p below 0.05 after adjustment for multiple comparisons was considered significant.

## Results

A total of 75 patients were enrolled in the study during the period June 2008 to March 2012; 39 with sepsis, 25 with severe sepsis, and 11 with septic shock; their APACHE II score was 15.6 ± 8.1. Their demographic and clinical characteristics are shown at Table 1. The most common causes of sepsis were acute pyelonephritis, intra-abdominal infections and community acquired pneumonia (CAP). Sixteen patients died due to sepsis (mortality 21.3%). All patients survived until day 3; 70 patients remained alive until day 7.

**Table 1:** **Demographic and clinical characteristics of patients**

	**Total (%)**
	75
Male/Female	39/36
Age (years, mean ± SD)	73.6 ± 1 7.3
APACHE II score (mean ± SD)	15.6 ± 8.1
Disease severity	
Sepsis	41 (54.7)
Severe Sepsis	25 (33.3)
Septic Shock	9 (12.0)
Type of infection	
Acute pyelonephritis	25 (33.3)
Intra-abdominal infections	16 (21.3)
Lung infections	14 (18.7)
Bloodstream infections	20 (26.7)
Bacterial isolate	
None	39 (52.0)
*Escherichia coli*	17 (22.7)
*Klebsiella pneumoniae*	7 (9.3)
*Acinetobacter baumannii*	3 (4.0)
*Proteus mirabilis*	2 (2.7)
*Pseudomonas aeruginosa*	2 (2.7)
*Enterococcus faecalis*	2 (2.7)
Other	3 (4.0)
28-day mortality	16 (21.3)

**Figure 1:**
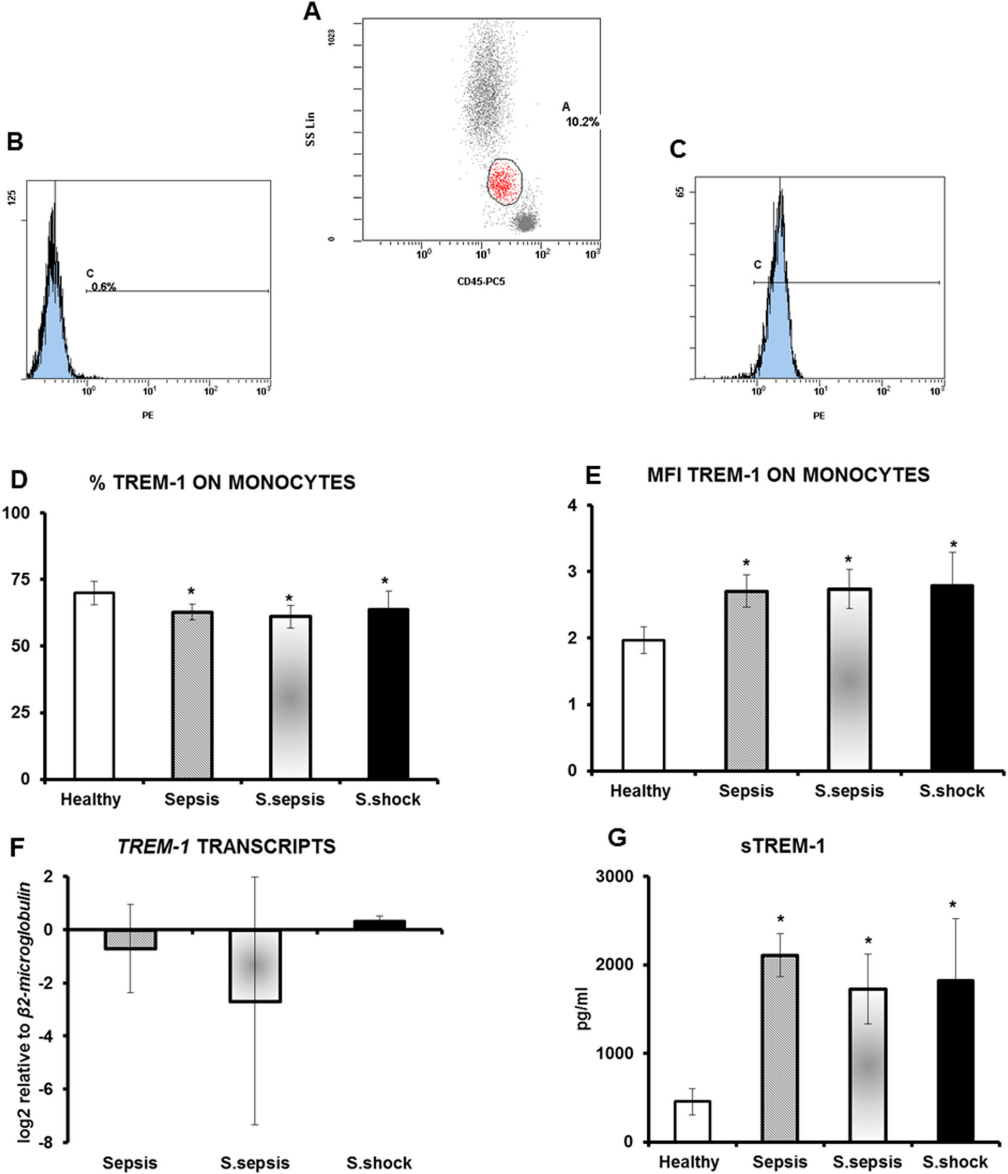
**Expression of TREM-1 on monocytes A) gating on monocytes; B) negative isotype anti-IgG1-PE control; C) staining with anti-TREM-1-PE; D) % expression of TREM-1 on monocytes of healthy volunteers (n = 10), of patients with sepsis (n = 41), of patients with severe sepsis (n = 25) and of patients with septic shock (n = 9); E) fluoresecence of TREM-1 on monocytes of healthy volunteers (n = 10), of patients with sepsis (n = 41), of patients with severe sepsis (n = 25) and of patients with septic shock (n = 9); F) relative transcripts of** ***TREM-1*** **in the cytoplasm monocytes of patients with sepsis (n = 12), of patients with severe sepsis (n = 20) and of patients with septic shock (n = 6); and G) circulating sTREM-1 of healthy volunteers (n = 10), of patients with sepsis (n = 41), of patients with severe sepsis (n = 25) and of patients with septic shock (n = 9).** Asterisks denote statistical significant differences compared to healthy volunteers.

At first the number of *TREM-1* transcripts, the expression of TREM-1 on monocytes and circulating levels of sTREM-1 of the first day of sampling were measured in relation with the stage of sepsis. No differences were found between sepsis, severe sepsis and septic shock (Figure 1). Reported results of flow cytometry were robust since the overall purity of the gated cells for monocytes was more than 99.1% (Additional file 1: Figure S1). The preparation of the samples through lysis of red blood cells did not affect expression of TREM-1 on monocytes (Additional file 2: Figure S2).

**Figure 2:**
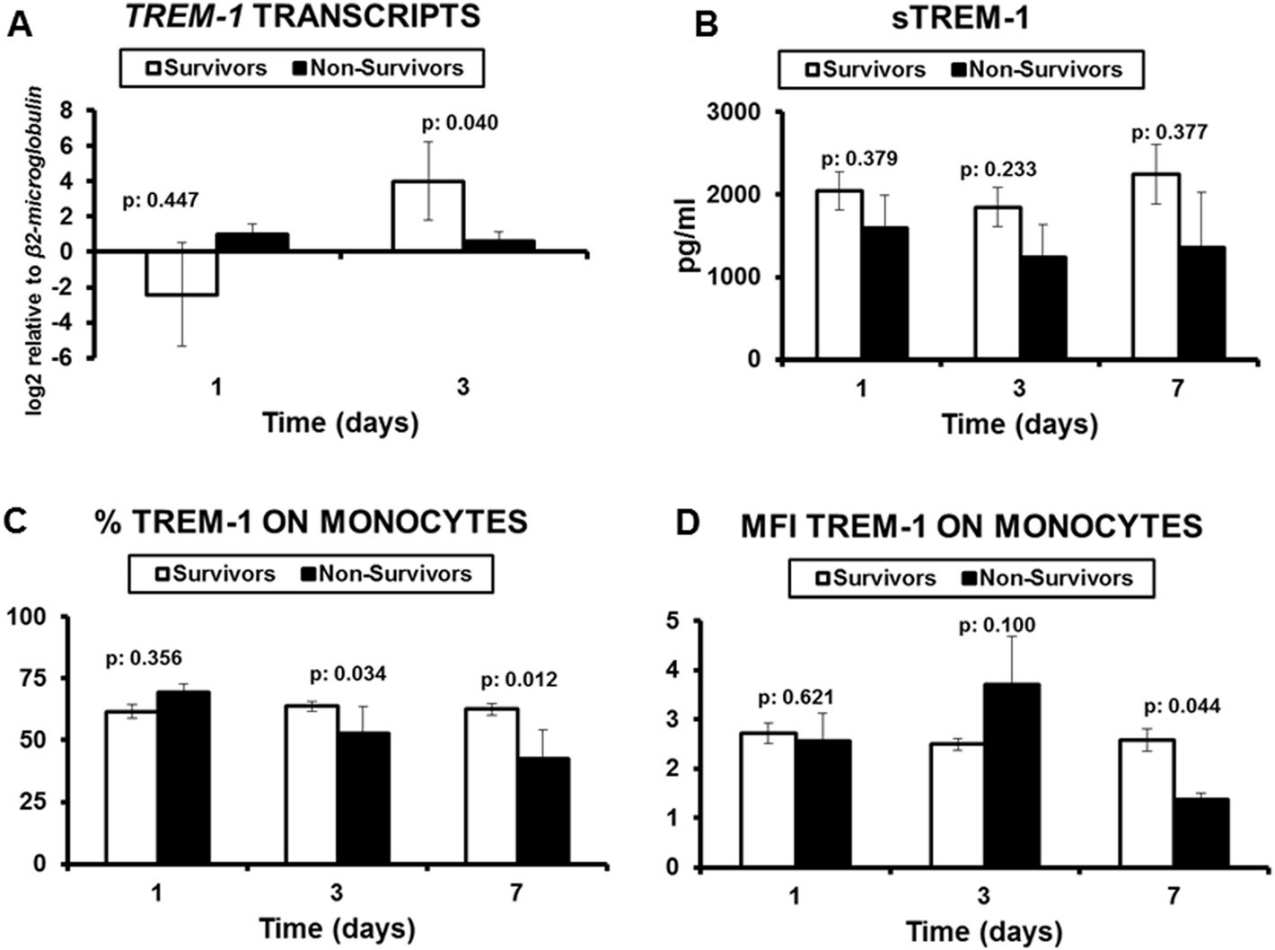
**Comparisons between survivors and non-survivors A)** ***TREM-1*** **transcripts on monocytes (n of survivors = 25; n of non-survivors = 13); B) circulating sTREM-1 on consecutive days (n of survivors = 59; n of non-survivors = 16); C) % expression of TREM-1 on circulating monocytes (n of survivors = 59; n of non-survivors = 16); and D) MFI expression of TREM-1 on circulating monocytes (n of survivors = 59; n of non-survivors = 16).** P values represent comparisons between survivors and non-survivors.

This lack of differences between the stages of sepsis prompted us to investigate the early kinetics of TREM-1 and *TREM-1*. To this end, expression of TREM-1 on monocytes, circulating sTREM-1 and *TREM-1* transcripts were compared on consecutive days between survivors and non-survivors (Figure 2). Results showed that gene expression of *TREM-1* was significantly greater in survivors on day 3 compared with non-survivors. On the same day, although circulating sTREM-1 did not differ between survivors and non-survivors, the % expression of TREM-1 on monocytes was significantly greater in survivors than in non-survivors. This difference was also found on day 7. Although no differences were observed between survivors on consecutive days, paired comparisons among non-survivors showed that circulating sTREM-1 was decreased from day 1 to day 3 (p: 0.011) and that expression of TREM-1 on monocytes was also decreased from day 1 to day 3 (p: 0.043).

**Figure 3:**
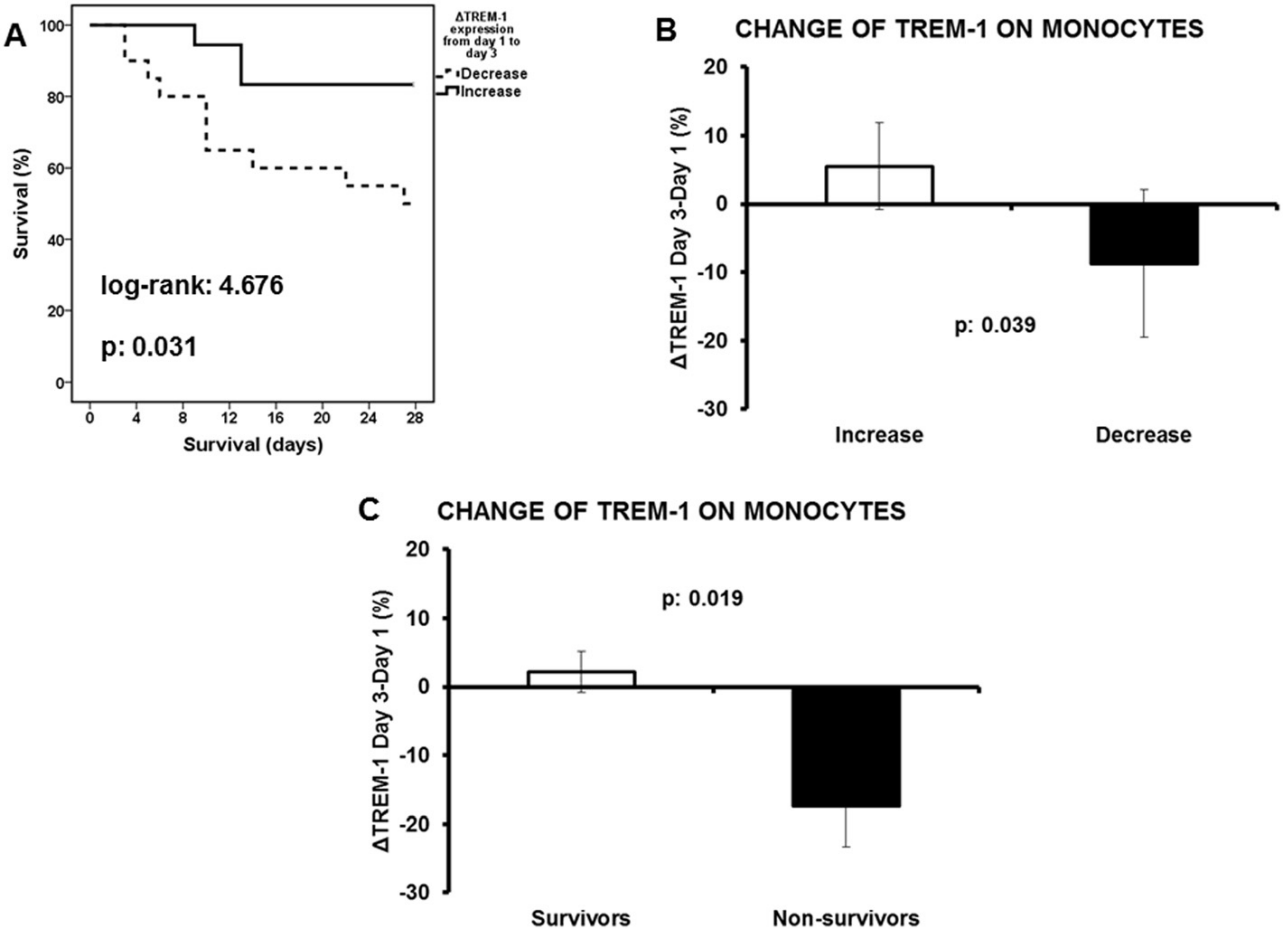
**Study primary endpoint: impact of** ***TREM-1*** **expression on final outcome A) Survival analysis comparing patients with decrease of** ***TREM-1*** **expression from day 1 to day 3 (n = 20) and patients with increase of** ***TREM-1*** **expression from day 1 to day 3 (n = 18); B) Respective comparisons of the change of the expression of TREM-1 on circulating monocytes are provided; C) Comparison of the change of TREM-1 expression on circulating monocytes between day 3 and day 1 between survivors (n = 59) and non-survivors (n = 16).** P values of comparisons are provided.

To investigate the primary endpoint, patients were divided into those whose gene expression of *TREM-1* on circulating monocytes increased from day 1 to day 3 and into those whose gene expression of *TREM-1* on circulating monocytes decreased from day 1 to day 3. Survival of patients with increase of gene expression was prolonged compared to those with decrease of gene expression (Figure 3A) what was accompanied by a reciprocal increase of the expression of the cellular receptor TREM-1 on circulating monocytes (Figure 3B). This was associated with an increase of the expression of TREM-1 on circulating monocytes of survivors from day 1 to day 3 (Figure 3C). It was found that 83.3% of patients with increased gene expression of *TREM-1* from day 1 to day 3 were administered appropriate antimicrobials compared to 76.9% of patients with decreased gene expression of *TREM-1* (p: 0.627).

## Discussion

TREM-1 regulates innate immunity and inflammatory responses to microbial products and its functional significance is underlined by the protective role demonstrated by TREM-1 blockade in animal models of septic shock [22]. This study sought to understand the complex expression of *TREM-1* on monocytes in sepsis. Within the first 24 hours from sepsis onset there was no significant difference in *TREM-1* gene expression, surface TREM-1 and circulating sTREM-1 between survivors and non-survivors. However, dramatic changes were found within the next 48 hours markedly related with final outcome; failure for increase of *TREM-1* gene expression was associated with decrease of surface TREM-1 on monocytes and of circulating sTREM-1. All these changes were linked with unfavorable outcome.

Given the significance of TREM-1 in the inflammatory cascade, its gene expression at sepsis onset has been previously investigated. The results of these studies are in agreement with a previous study in Greek patients and re-confirm that the gene expression of *TREM-1* at baseline is not different between sepsis, severe sepsis and septic shock [15]. Oku et al. showed that TREM-1 expression on the cell membranes of monocytes did not correlate with either serum IL-6 level or the count of monocyte in septic patients [23], and the same finding was demonstrated by Tao et al. [24]. However, none of these investigators explored the over-time changes of the gene expression of *TREM-1* and its significance for the final outcome. To our knowledge, this is the first study that attempted to define the temporal pattern of *TREM-1* gene expression on circulating monocytes and its liaison with final outcome. Although this finding was not confounded by the appropriateness of antimicrobial treatment, it should bear in mind that sepsis is a heterogeneous process where host related factors and therapeutic interventions impact greatly on final outcome.

The temporal variation in gene expression during sepsis has received much attention lately. Marked variability in differential gene expression has been observed between time points and between patients, revealing the dynamic character of gene expression during the evolution of sepsis [25]. Given the central role of monocyte in the innate immune response, monocyte functionality has been intensely investigated. Monocyte gene expression analysis has been carried out [26], providing interesting results [27], and highlighting the significance of gene kinetics. The elucidation of this kinetics could allow a closer follow up and might offer new insight in the etiologic management of the septic syndrome.

The current concept of sepsis is described by initial increases in inflammatory mediators, but with time and as sepsis persists, a shift to an anti-inflammatory immunosuppressive state prevails [28]. A recent postmortem study of spleen and lung tissues of patients dying from sepsis revealed flow cytometric, and immunohistochemical findings consistent with systemic immunosuppression, compared to non-septic controls [29]. Signs of immunoparalysis were also evident in a clinical model of sepsis caused by LPS infusion in healthy volunteers and partially reversed by IFN-γ [30]. Current results suggest that decrease of gene expression of *TREM-1* encoding for the pro-inflammatory receptor TREM-1 may be a component of sepsis-induced immunosuppression and that this is associated with decrease of TREM-1 molecules on circulating monocytes, with decrease of circulating sTREM-1 and with unfavorable outcome. On the contrary, when gene expression of *TREM-1* is increased, surface TREM-1 on monocytes and circulating sTREM-1 remain at stable levels and this is associated favorable outcome probably as a result of gene translation. Findings strongly emphasize the important role of the kinetics of TREM-1 at the stage of immunoparalysis [8]. Whether this could be translated to a therapeutic approach on a clinical level remains theoretical and needs further investigation.

Regarding sTREM-1, our current results are in line with previous results from our group demonstrating that serum kinetics of sTREM-1 did not follow changes of the expression of *TREM-1* [15]. This could suggest that the levels of sTREM-1 are determined by more complex mechanisms than simple shedding of the membrane receptor.

## Conclusions

The results of the present study demonstrate a major role of TREM-1 in acute septic process; a decrease in monocyte gene *TREM*-1 expression during the first 3 days of sepsis is associated with worse outcome. Whether these changes in *TREM*-1 expression have a causative relation with outcome, or represent simply severity and prognostic marker is not clear. A deeper understanding of pathophysiology is necessary for proper immunological monitoring and potentially effective interventions.

## Additional files

Additional file 1: Figure S1. Purity of studied monocytes**.** Gating on monocytes is shown for three different patients (panels A, E and I). Staining for CD3 (respective panels B, F and J), CD19 (respective panels C, G and K) and CD14 (respective panels D, H and L) on this gate is provided. Additional file 2: Figure S2. Effect of red blood cell lysis on measurement of TREM-1 expression on monocytes. An example of one patient is shown: A) Flow cytometer analysis of non-lysed blood cells stained with anti-CD45; monocytes are gated. B) Flow cytometer analysis of TREM-1 expression of gated monocytes in panel A. C) Flow cytometer analysis of blood cells following lysis of red blood cells stained with anti-CD45; monocytes are gated. D) Flow cytometer analysis of TREM-1 expression of gated monocytes in panel C. MFI: mean fluorescence intensity.

## Abbreviations

APACHE: Acute physiology and chronic health evaluation
CAP: Community-acquired pneumonia
HAP: Hospital-acquired pneumonia
LPS: Lipopolysaccharide
MFI: Mean fluorescence intensity
sTREM-1: Soluble triggering receptor expressed on myeloid cells-1
TREM-1: Triggering receptor expressed on myeloid cells-1
VAP: Ventilator-associated pneumonia
