# Supplementary figures and images for: Early changes of the kinetics of monocyte trem-1 reflect final outcome in human sepsis
Source: BMC Immunol. 2014 Dec 23;15:585. doi: 10.1186/s12865-014-0063-y (PMC4335537; doi:10.1186/s12865-014-0063-y)

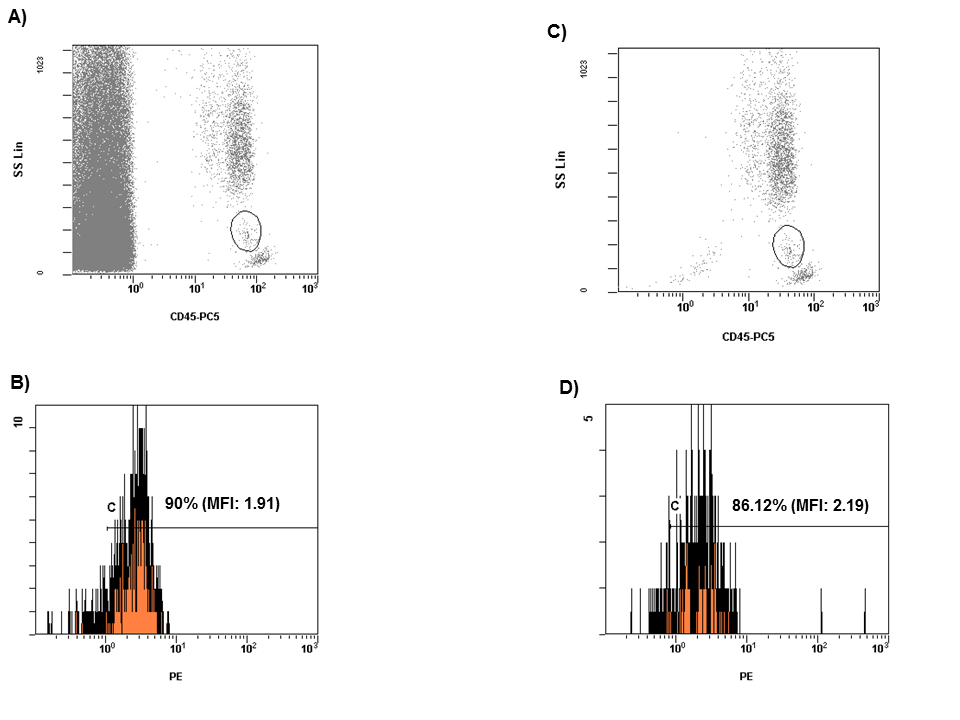

Supplement: Additional file 1: Figure S1. — Purity of studied monocytes. Gating on monocytes is shown for three different patients (panels A, E and I). Staining for CD3 (respective panels B, F and J), CD19 (respective panels C, G and K) and CD14 (respective panels D, H and L) on this gate is provided. [file 12865_2014_63_MOESM1_ESM.tiff]

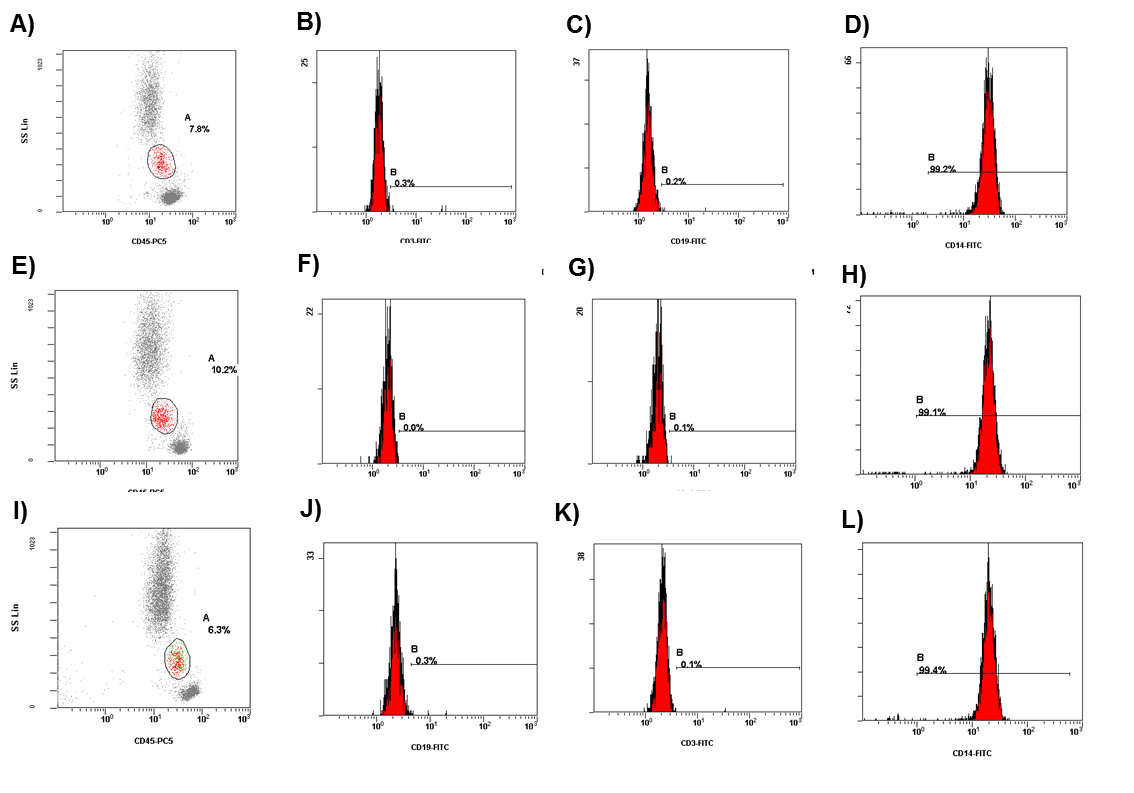

Supplement: Additional file 2: Figure S2. — Effect of red blood cell lysis on measurement of TREM-1 expression on monocytes. An example of one patient is shown: A) Flow cytometer analysis of non-lysed blood cells stained with anti-CD45; monocytes are gated. B) Flow cytometer analysis of TREM-1 expression of gated monocytes in panel A. C) Flow cytometer analysis of blood cells following lysis of red blood cells stained with anti-CD45; monocytes are gated. D) Flow cytometer analysis of TREM-1 expression of gated monocytes in panel C. MFI: mean fluorescence intensity. [file 12865_2014_63_MOESM2_ESM.tiff]
